# COVID-19 Rebound in Nirmatrelvir Plus Ritonavir Treatment and Control Groups: Prospective Cohort Study

**DOI:** 10.2196/80263

**Published:** 2026-06-02

**Authors:** Jacqueline K Kueper, Kalyani Kottilil, Giorgio Quer, Danielle C Chiang, Emily G Spencer, Jyothi Purushotham, Edward Ramos, Leila Roumani, Kristian G Andersen, Eric J Topol, Jay A Pandit, Michael J Mina

**Affiliations:** 1 Scripps Research Translational Institute, Scripps Research Institute, 10550 North Torrey Pines Road, La Jolla, CA, 92037, United States, 1 858-784-1000; 2 Department of Immunology and Microbiology, Scripps Research Institute, La Jolla, CA, United States; 3 Care Evolution, Ann Arbor, MI, United States; 4 eMed, Miami, FL, United States; 5 Immune Observatory, Boston, MA, United States

**Keywords:** COVID-19, COVID-19 rebound, nirmatrelvir plus ritonavir, symptom rebound, viral rebound

## Abstract

**Background:**

Observation of COVID-19 rebound after nirmatrelvir plus ritonavir (NPR) has driven important questions surrounding one of the only direct-acting antiviral treatments for COVID-19.

**Objective:**

The objective of this study was to examine the epidemiology of COVID-19 rebound among COVID-19–positive outpatients in the United States who independently decided whether or not to take NPR.

**Methods:**

This prospective, decentralized observational cohort study was conducted from August 2022 through December 2023 and included frequent proctored COVID-19 rapid antigen tests and self-report symptom surveys for 15 days. The primary outcome was the incidence of viral and symptom rebound. Secondary outcomes included time to initial viral and symptom clearance, rebound probability among patients who cleared by day 15, and symptom frequency.

**Results:**

Of 917 consenting participants, 669 (73%) were eligible for inclusion in the analysis (n=443, 66% in the NPR group; n=226, 34% in the control group). The mean age was 46.1 (SD 12.9) years, 62.6% (n=419) of participants were female, and 49.2% (n=329) had at least one preexisting condition. Overall, 15-day cumulative incidence was higher in the NPR group than the control group for both viral (70/443, 15.8% vs 12/226, 5.3%) and symptom (73/443, 16.5% vs 19/226, 8.4%) rebound. Time to initial viral and symptom clearance was similar between groups, and among those who experienced clearance by day 15, the probability of viral rebound (NPR: 19.1%, 95% CI 15.1%-24.0% vs control: 7%, 95% CI 4.0%-12.6%; *P*<.001) and symptom rebound (NPR: 47.7%, 95% CI 36.1%-60.8% vs control: 16.9%, 95% CI 10.9%-25.7%; *P*<.001) was higher in the NPR group than the control group.

**Conclusions:**

This study demonstrates that while COVID-19 rebound occurs in both NPR-treated and untreated outpatients, the incidence is higher in the NPR group.

## Introduction

In the United States, more than 1 million deaths and approximately 7 million hospitalizations reflect the toll of SARS-CoV-2 [1]. After initial emergency use authorization in December 2021 [2], nirmatrelvir plus ritonavir (NPR; Paxlovid) became the first fully approved oral antiviral treatment by the US Food and Drug Administration in May 2023 [3]. US COVID-19 treatment guidelines align on a strong recommendation for a 5-day course of NPR in outpatients at high risk of progression to severe COVID-19 [4-6].

The guidance in favor of NPR is largely based on studies showing significant protective effects against hospitalization and death for high-risk outpatients, with little evidence of serious side effects, across changing COVID-19 variants and baseline population risk levels [4-7]. Despite this, it has been estimated that less than a quarter of eligible patients use NPR, potentially missing opportunities to further reduce serious illness and death rates [8-10]. Hesitation in NPR prescription may be associated with anecdotal reports and poor knowledge about COVID-19 rebound [8,11].

COVID-19 rebound is the resurgence in viral load and/or COVID-19 symptoms after initial recovery. This phenomenon first gained attention as a secondary finding in early studies testing NPR effectiveness against severe disease outcomes, although it also occurs in untreated individuals [12,13]. In direct response to the need for further investigation, we opened a prospective observational cohort in 2022 (COVID-19 Rebound Study) to evaluate the epidemiology of viral and symptom rebound differences in NPR treatment and control individuals based on high-frequency testing and symptom surveys [14]. Preliminary results found higher than previously reported COVID-19 viral and symptom rebound in both NPR and control groups [14]. While the amount of research on rebound is increasing, evidence supporting a potential association with NPR treatment is still relatively weak, relying on retrospective chart reviews or secondary analyses [15].

This paper describes the final results of the COVID-19 Rebound Study, expanding upon our previously published preliminary analysis [14], with a larger sample size and extended enrollment period to evaluate the epidemiology of COVID-19 rebound in participants who were clinically eligible for NPR and who did or did not decide to take NPR.

## Methods

### Study Design and Recruitment

This decentralized, prospective observational study was completed via collaboration between the Scripps Research Translational Institute (SRTI) and eMed, which implemented the National Institutes of Health Home Test to Treat Program, a 24/7 virtual health platform that provides proctor-guided at-home testing with public health reporting through a digital Clinical Laboratory Improvement Amendments (CLIA)–waived laboratory. Eligibility criteria included a positive rapid antigen test for COVID-19 within 48 hours prior to study enrollment, residing within the United States, speaking English, and aged at least 18 years. eMed users who tested positive were offered NPR at the discretion of an eMed provider. NPR was the only COVID-19 oral antiviral therapy offered through the eMed platform. There were two study recruitment cohorts: (1) from August 2022 through December 2023, eligible eMed users that tested positive for COVID-19 through an eMed telehealth appointment were invited via follow-up email, text, and/or phone call; and (2) from August 2023 through December 2023, social media campaigns and SRTI blog posts invited people who self-tested positive to consider participation. Participation required a virtual meeting with an eMed provider to confirm a positive COVID-19 rapid antigen test and for further eligibility screening.

### Study Procedures

Interested individuals were sent to a landing page with more information on the study, an eligibility screener survey, and e-consent forms. Upon providing consent, participants reported baseline information (self-reported demographic characteristics, preexisting conditions, and vaccination history) and completed a symptom survey prior to requesting a study kit. Study kits were shipped overnight and included detailed instructions for the 15-day acute study phase and eMed live-proctor–enabled COVID-19 test-to-treat rapid antigen test kits (containing Abbott BinaxNOW Home tests). Participants who enrolled after August 2023 (cohort 2) were additionally sent 2 at-home blood collection devices with biohazard shipping materials. Cohort 2 was instructed to return blood samples and testing swabs to SRTI for serological analyses and viral genome sequencing. Results from blood sampling and sequencing are forthcoming in a future report.

Participants were asked to complete a survey (capturing symptoms and NPR use) and a proctored eMed COVID-19 rapid antigen test on days 2, 5, 7, 9, 11, 13, and 15 from enrollment (acute study phase). To complete a rapid antigen COVID-19 test, participants were instructed to scan a QR code on their test box to initiate a telehealth proctored visit where they were guided through the testing procedure and test results validated. If a participant tested positive during the visit and had not already received care either in person or through a telemedicine visit, they were offered an optional eMed telemedicine visit that could provide a prescription for a 5-day course of NPR (provided under the brand name Paxlovid). Postacute COVID-19 symptom surveys were sent to all participants at 1-, 3-, and 6-months after enrollment, with results forthcoming. Participants received up to 3 notification reminders per task using their preferred mode of communication (email, text, and phone) unless they chose to withdraw early. Research Electronic Data Capture (REDCap) tools were used for survey and notification administration [16,17]. Participants were offered an incentive of electronic gift cards totaling up to US $200, which were issued over the study period according to completion of required tasks.

### Outcomes

The primary outcomes were cumulative incidence of viral and symptom rebound in NPR and control groups within 15 days of enrollment. *Viral rebound* was defined as a positive rapid antigen test after previously testing negative. *Symptom rebound* was defined as reporting at least one symptom on a survey after reporting zero symptoms on a previous survey. Secondary outcomes included time from enrollment to initial negative rapid antigen test and to symptom resolution (viral and symptom clearance, respectively), and rebound probability among patients with clearance by day 15. Exploratory outcomes included comparing characteristics of rebound and nonrebound groups and symptom frequency during the acute study phase. Reported symptoms were aggregated into binary categories of systemic, respiratory, gastrointestinal, neurological, and other symptoms.

### Statistical Analyses

#### Treatment Groups

Participants who reported their independent decision to take at least one NPR dose were classified as the NPR group, and remaining participants were classified as the control group. Participants were excluded if they did not complete the baseline survey or at least 2 follow-up surveys, or if they had missing data precluding ascertainment of rebound. Participants reporting no symptoms at baseline (n=5) were excluded from symptom rebound analyses.

#### Main Analyses

Cumulative incidence was calculated as the number of rebound events across the 15-day acute study phase divided by the number of eligible participants. Kaplan-Meier survival curves were generated and stratified by NPR and control groups to compare timing of rebound since study enrollment, of viral and symptom clearance (time from positive test or symptoms to first negative test or no symptoms), and of rebound probability among patients who cleared by day 15 (time from negative test or no symptoms to subsequent positive test or symptoms). In each time-to-event analysis, participants were censored at the time of missing more than 2 consecutive surveys or rapid antigen tests or after day 15 if they did not have an event. The 95% CIs were calculated using the Greenwood exponential formula.

#### Secondary Analyses

Due to potential differences between participants recruited via the eMed platform and social media, we conducted sensitivity analyses comparing baseline characteristics and outcomes between recruitment cohorts. We additionally conducted 5 secondary analyses. First, we compared baseline participant characteristics, rebound incidence, and symptom frequency between the 2 cohorts (ie, recruited before or after August 2023). Second, we assessed sensitivity of cumulative incidence estimates to different censoring (restricted to those with no missing data or those with at least 3 surveys before missing any data), time (11- vs 15-day estimates), and amount of NPR use (none, 1‐4 days, or 5 days) criteria. Participant characteristics and frequency of symptom surveys were additionally compared between groups defined by the amount of NPR use reported. Third, we adjusted rebound incidence comparisons for baseline characteristics using Cox proportional hazards regression models. Separate models were run separately for viral and symptom rebound and included covariates for age, number of vaccines, sex, race, and preexisting conditions. As compared with the main participant characteristic table groupings, additional data cleaning was performed to collapse small cell counts, including grouping of cardiovascular conditions (heart disease, heart failure, and high blood pressure), grouping of lung conditions (asthma, chronic bronchitis, chronic obstructive pulmonary disease, emphysema, and other lung conditions), removing the single intersex participant, and combining race categories with fewer than 3 participants into the other category. Fourth, we examined congruence between symptom and test positivity by counting the number of person-days (ie, unique participant surveys) where symptoms and tests were both positive, negative, or opposite of each other. This was done for all acute phase days and for the subset of person-days where a participant was testing positive in an episode of viral test positivity. Fifth, we compared baseline characteristics of participants who were and were not eligible for inclusion in analyses.

Analyses were done with Python (version 3.8; Python Software Foundation). Between-group statistical comparisons of baseline characteristics and symptoms were performed using the TableOne package with 2-sided chi-square tests for categorical variables and *t* tests for continuous variables, alpha level .05 [18]. The Benjamini-Hochberg procedure to control for false discovery rate was applied across multiple tests. Time-to-event analyses were performed using the lifelines package [19]. We followed STROBE (Strengthening the Reporting of Observational Studies in Epidemiology) reporting guidelines [20] (Multimedia Appendix 1).

### Ethical Considerations

The study was approved by the Scripps Institutional Review Board (22‐7978). All participants provided electronic informed consent. SRTI performed safety oversight.

## Results

### Study Population

**Figure 1.**
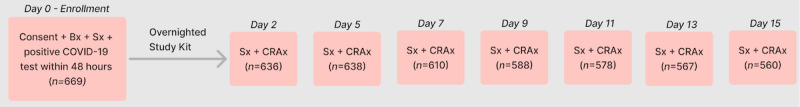
Study flow. Study task completion for the analysis sample across days since enrollment. Bx: baseline survey; CRAx: study provided COVID-19 rapid antigen test; n: number of participants meeting analysis eligibility criteria that completed the survey; Sx: symptom survey.

Of 917 consented participants, 669 (73%) were eligible for inclusion in analyses (Figure 1); of these, 443 (66.2%) reported taking at least one dose of NPR (NPR group) and 226 (33.8%) did not (control group). Within the NPR group (n=443), 197 (44.5%) reported taking all 5 days of their NPR prescription. The average age was 46.1 (SD 12.9) years, and 62.6% (419/669) of participants were female. The most common preexisting condition was high blood pressure (132/669, 19.7%), and half of participants (340/669, 50.8%) did not report any preexisting conditions. While sex was similar between groups, there were differences in age (56/67, 83.6% of those older than 65 years opting to take NPR compared with 189/258, 73.3% of those aged 45-65 years and 198/344, 57.6% of those aged 18-44 years), race (351/491, 71.5% of White participants opting to take NPR compared with 85/168, 50.3% of non-White participants), and the presence of preexisting conditions (Table 1). The proportion of participants in the NPR group was higher in the first cohort (181/251, 72.1%) than the second cohort (262/418, 62.7%; Table S1 in Multimedia Appendix 1).

**Table 1.** Participant characteristics by treatment group^a^.

			Overall (n=669)	Control (n=226)	NPR^b^ (n=443)	*P* value
Age (years), n (%)						.005
18‐44			344 (51.4)	146 (64.6)	198 (44.7)	
45‐64			258 (38.6)	69 (30.5)	189 (42.7)	
≥65			67 (10)	11 (4.9)	56 (12.6)	
Sex, n (%)						.87
Female			419 (62.6)	143 (63.3)	276 (62.3)	
Male			249 (37.2)	83 (36.7)	166 (37.5)	
Intersex			1 (0.1)	0 (0)	1 (0.2)	
Race, n (%)						.005
American Indian or Alaska Native			2 (0.3)	1 (0.4)	1 (0.2)	
Asian			42 (6.3)	19 (8.4)	23 (5.2)	
Black, African American, or African			41 (6.1)	20 (8.8)	21 (4.7)	
Hispanic, Latino, or Spanish			47 (7)	27 (11.9)	20 (4.5)	
White			491 (73.4)	140 (61.9)	351 (79.2)	
Multiple			33 (4.9)	15 (6.6)	18 (4.1)	
Other			3 (0.4)	1 (0.4)	2 (0.4)	
Declined to answer			10 (1.5)	3 (1.3)	7 (1.6)	
At least one COVID-19 vaccine, n (%)			637 (95.2)	208 (92.0)	429 (96.8)	.04
Preexisting conditions, n (%)						
Asthma			117 (17.5)	34 (15)	83 (18.7)	.52
Autoimmune condition			63 (9.4)	17 (7.5)	46 (10.4)	.52
Cancer			35 (5.2)	5 (2.2)	30 (6.8)	.06
Chronic bronchitis			10 (1.5)	3 (1.3)	7 (1.6)	>.99
Chronic obstructive pulmonary disease			3 (0.4)	3 (1.3)	0 (0)	.10
Diabetes			44 (6.6)	10 (4.4)	34 (7.7)	.34
Emphysema			1 (0.1)	1 (0.4)	0 (0)	.54
Heart disease			21 (3.1)	5 (2.2)	16 (3.6)	.56
Heart failure			3 (0.4)	1 (0.4)	2 (0.5)	>.99
High blood pressure			132 (19.7)	40 (17.7)	92 (20.8)	.56
Other lung condition			7 (1)	1 (0.4)	6 (1.4)	.56
None			340 (50.8)	137 (60.6)	203 (45.8)	.005
15-day COVID-19 rebound incidence, n (%)						
Viral test rebound			82 (12.3)	12 (5.3)	70 (15.8)	.002
Symptom rebound			92 (13.8)	19 (8.4)	73 (16.5)	.006
Acute phase symptom surveys, mean (SD)						
Systemic symptoms			3.5 (2.4)	3.2 (2.2)	3.7 (2.4)	.04
Respiratory symptoms			4.7 (2.4)	4.5 (2.4)	4.8 (2.4)	.13
Gastrointestinal symptoms			1.0 (1.4)	0.9 (1.3)	1.1 (1.5)	.06
Neurological symptoms			0.4 (1.0)	0.3 (0.7)	0.5 (1.2)	.04
Other symptoms			0.4 (0.9)	0.3 (0.8)	0.4 (1.0)	.06

a The acute phase symptom category measures are cumulative and represent the number of survey days across the 15-day acute study phase during which participants reported experiencing each symptom type (maximum 8 surveys).

b NPR: nirmatrelvir plus ritonavir.

### Primary Outcome: Rebound Incidence

**Figure 2.**
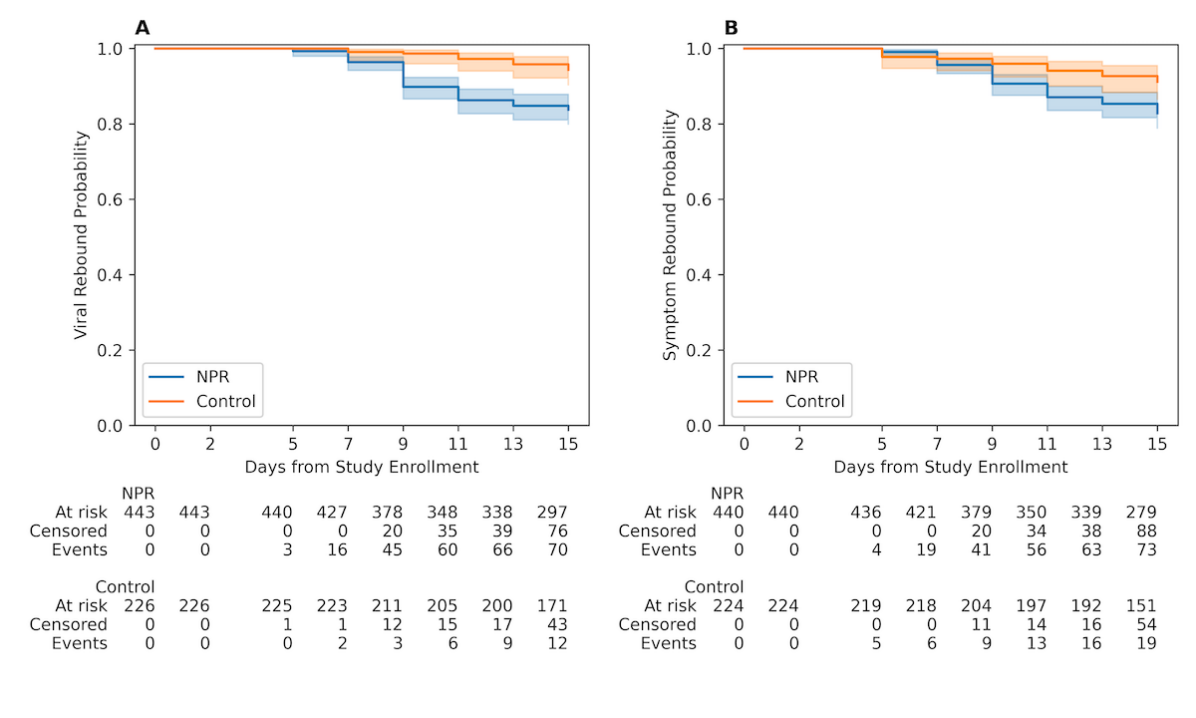
Kaplan-Meier curves between nirmatrelvir plus ritonavir (NPR) and control groups for overall rebound risk from baseline. Curves present the probability of events for our primary outcome measure of overall (A) viral and (B) symptom rebound risk since study enrollment.

The 15-day cumulative viral rebound incidence was 12.3% (82/669) overall (NPR: 70/443, 15.8% and control: 12/226, 5.3%). The 15-day cumulative symptom rebound incidence was 13.8% (92/664) overall (NPR: 73/443, 16.5% and control: 19/226, 8.4%). Kaplan-Meier curves showed significantly increased probability of viral test rebound by day 15 of 16.2% (95% CI 13.1%‐20.1%) for the NPR group and 5.6% (95% CI 3.2%‐9.6%) for the control group (Figure 2; log-rank *P*<.001). Probability of symptom rebound by day 15 was 17.2% (95% CI 13.9%‐21.1%) and 8.8% (95% CI 5.7%‐13.4%) for NPR and control groups, respectively (Figure 2; log-rank *P*=.004). Secondary analyses examining incidence across different eligibility and timing criteria (Table S2 in Multimedia Appendix 1), whether a partial (1‐4 days) or full (5 days) prescription of NPR was taken (Table S3 in Multimedia Appendix 1), and adjusted for baseline demographic and clinical characteristics using Cox proportional hazards regression (Tables S4 and S5 in Multimedia Appendix 1) did not change conclusions.

### Secondary Outcomes

#### Initial Viral and Symptom Clearance

**Figure 3.**
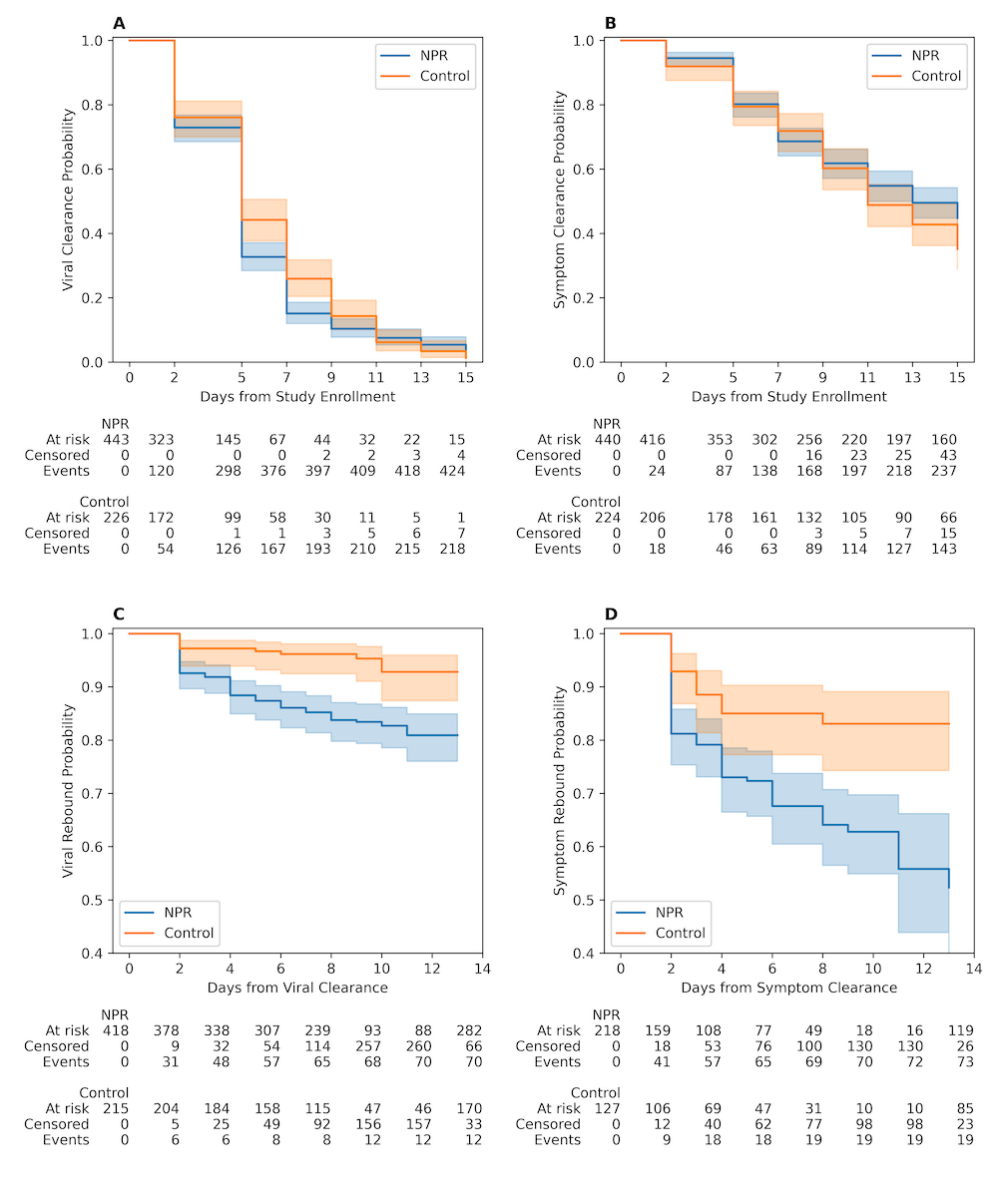
Kaplan-Meier curves between nirmatrelvir plus ritonavir (NPR) and control groups for clearance and clearance-dependent rebound risk. Curves present the probability of events for our secondary outcomes of initial clearance of (A) virus test positivity or (B) resolution of symptoms and (C and D) risk of rebound following initial clearance or resolution.

Time from enrollment to initial viral clearance was similar in the NPR and control groups (Figure 3; log-rank *P*=.11). Among the 669 participants, 642 (96%) who experienced viral clearance, the mean time to clearance was 5.4 (SD 2.9) days (NPR: mean 5.2, SD 2.8 days and control: mean 5.9, SD 3.1 days; Figure S1 in Multimedia Appendix 1). A total of 16 (2.4%) participants (NPR: 15/16, 93.7% and control: 1/16, 6.3%) never tested negative throughout the 15-day acute study phase. Another 11 (1.6%) participants (NPR: 4/11, 36.4% and control: 7/11, 63.6%) had unknown clearance timing due to too many sequentially missing tests.

Time to initial symptom clearance tended to take longer than viral clearance but was also similar between the NPR and control groups (Figure 3; log-rank *P*=.06). Of the 664 participants who reported at least one symptom at baseline, 380 (57.2%) reported symptom clearance in a mean of 8.1 (SD 3.8) days (NPR: mean 7.9, SD 3.7 days and control: mean 8.5, SD 4.0 days; Figure S1 in Multimedia Appendix 1). There were 226 (34%) participants (NPR: 160/226, 70.8% and control: 66/226, 29.2%) who did not report symptom clearance by day 15. An additional 58 (8.7%) participants (NPR: 43/58, 74.1% and control: 15/58, 25.9%) had too many sequentially missing symptom surveys to determine clearance timing.

#### Rebound Following Initial Clearance

The primary outcome was rebound incidence among all participants under evaluation, regardless of whether they experienced initial viral or symptom clearance. Therefore, the previous evaluation provided an estimate of risk for a patient group that did not yet know if they would clear the virus or symptoms in time to become “eligible” to experience a rebound event within the 15-day acute phase. Thus, we also explored the critical question of rebound probability among patients who cleared by day 15.

Among the 633 participants who experienced initial viral clearance before day 15, the risk of viral rebound by day 15 was 19.1% (95% CI 15.1%‐24.0%) for the NPR group compared with 7.2% (95% CI 4.0%‐12.6%) for the control group (Figure 3; log-rank *P*<.001). The mean number of days from initial viral clearance to rebound positive test was 4.3 (SD 2.7) overall (NPR: mean 4.1, SD 2.5 days and control: 5.2, SD 3.5 days; Figure S2 in Multimedia Appendix 1). Almost one-third (139/418, 30%) of viral rebound cases in the NPR group lasted 2 days or less, while 17.1% (71/418), 11.4% (48/418), and 2.9% (12/418) lasted 2 to 4, 4 to 6, and 6 to 8 days, respectively (Figure S3 in Multimedia Appendix 1). A total of 38.6% (27/70) NPR and 3.6% (4/110) control group viral rebound cases did not reclear prior to the end of the acute phase follow-up or when they were censored for missing tests.

Among the 345 participants who experienced initial symptom clearance before day 15, the risk of symptom rebound by day 15 was 47.7% (95% CI 36.1%‐60.8%) for the NPR compared with 16.9% (95% CI 10.9%‐25.7%) for the control group (Figure 3; log-rank *P*<.001). The mean number of days from initial symptom resolution to rebound was 3.5 (SD 2.3) overall (NPR: mean 3.6, SD 2.5 days and control: mean 3.0, SD 1.4 days; Figure S2 in Multimedia Appendix 1). The duration of rebound symptoms is presented in Figure S3 in Multimedia Appendix 1, with 54.8% (40/73) of NPR and 47.4% (9/19) of control group symptom rebound cases not resolved by day 15 or when they were censored.

### Exploratory Analyses

#### Characteristics of the Rebound and Nonrebound Groups

Table 2 compares characteristics of participants that did and did not experience each type of rebound. Of the 669 participants, there were 26 (3.9%) who experienced both viral and symptom rebound.

**Table 2.** Participant characteristics by viral and symptom test rebound^a^.

		No viral rebound (n=587)	Viral rebound (n=82)	*P* value	No symptom rebound (n=577)	Symptom rebound (n=92)	*P* value
Age (years), n (%)				.02			.59
18‐44		307 (52.3)	37 (45.1)		298 (51.6)	46 (50)	
45‐64		232 (39.5)	26 (31.7)		225 (39)	33 (35.9)	
≥65		48 (8.2)	19 (23.2)		54 (9.4)	13 (14.1)	
Sex, n (%)				>.99			.26
Female		366 (62.4)	53 (64.6)		368 (63.8)	51 (55.4)	
Male		220 (37.5)	29 (35.4)		209 (36.2)	40 (43.5)	
Intersex		1 (0.2)	0 (0)		0 (0)	1 (1.1)	
Race, n (%)				>.99			.50
American Indian or Alaska Native		2 (0.3)	0 (0)		2 (0.3)	0 (0)	
Asian		38 (6.5)	4 (4.9)		39 (6.8)	3 (3.3)	
Black, African American, or African		37 (6.3)	4 (4.9)		29 (5)	12 (13)	
Hispanic, Latino, or Spanish		42 (7.2)	5 (6.1)		44 (7.6)	3 (3.3)	
White		425 (72.4)	66 (80.5)		420 (72.8)	71 (77.2)	
Multiple		31 (5.3)	2 (2.4)		31 (5.4)	2 (2.2)	
Declined to answer		9 (1.5)	1 (1.2)		9 (1.6)	1 (1.1)	
Other		3 (0.5)	0 (0)		3 (0.5)	0 (0)	
At least one COVID-19 vaccine, n (%)		555 (94.5)	82 (100)	.15	548 (95)	89 (96.7)	.65
Preexisting conditions, n (%)							
Asthma		105 (17.9)	12 (14.6)	>.99	104 (18)	13 (14.1)	.59
Autoimmune condition		55 (9.4)	8 (9.8)	>.99	57 (9.9)	6 (6.5)	.59
Cancer		26 (4.4)	9 (11)	.15	29 (5)	6 (6.5)	.65
Chronic bronchitis		9 (1.5)	1 (1.2)	>.99	10 (1.7)	0 (0)	.59
COPD^b^		3 (0.5)	0 (0)	>.99	2 (0.3)	1 (1.1)	.59
Diabetes		36 (6.1)	8 (9.8)	.65	35 (6.1)	9 (9.8)	.59
Emphysema		1 (0.2)	0 (0)	>.99	1 (0.2)	0 (0)	>.99
Heart disease		16 (2.7)	5 (6.1)	.59	20 (3.5)	1 (1.1)	.59
Heart failure		2 (0.3)	1 (1.2)	.65	2 (0.3)	1 (1.1)	.59
High blood pressure		116 (19.8)	16 (19.5)	>.99	111 (19.2)	21 (22.8)	.63
Other lung condition		5 (0.9)	2 (2.4)	.59	5 (0.9)	2 (2.2)	.59
None		304 (51.8)	36 (43.9)	.59	298 (51.6)	42 (45.7)	.59
Acute phase symptom surveys, mean (SD)							
Systemic symptoms		3.5 (2.3)	3.9 (2.6)	.37	3.6 (2.4)	2.7 (1.9)	.005
Respiratory symptoms		4.5 (2.4)	5.6 (2.1)	.005	4.7 (2.5)	4.2 (1.8)	.03
Gastrointestinal symptoms		1.0 (1.4)	1.2 (1.7)	.37	1.1 (1.5)	0.8 (1.0)	.03
Neurological symptoms		0.4 (1.1)	0.3 (0.6)	.37	0.4 (1.1)	0.3 (0.7)	.46
Other symptoms		0.3 (0.9)	0.4 (1.1)	.51	0.4 (0.9)	0.3 (0.9)	.83

a The acute phase symptom category measures are cumulative and represent the number of survey days across the 15-day acute study phase during which participants reported experiencing each symptom type (maximum 8 surveys).

b COPD: chronic obstructive pulmonary disease.

#### Symptom Frequencies

Respiratory symptoms were the most commonly reported symptoms overall, with a mean number of 4.8 (SD 2.4) surveys in the NPR group and 4.5 (SD 2.4) surveys in the control group (*P=*.13; Table 1). The viral rebound group tended to report slightly more surveys with symptoms than those without viral rebound, with respiratory symptoms being the only statistically significant difference (*P*=.005; Table 2).

## Discussion

### Principal Findings

This COVID-19 Rebound Study found that participants who independently decided to take at least one dose of NPR had triple the 15-day cumulative incidence of viral test rebound and double the cumulative incidence of symptom rebound than the control group. These findings persisted in our secondary analysis after adjusting for baseline characteristics that may confound the association between NPR and COVID-19 rebound. Furthermore, when restricting the analysis to participants who achieved initial clearance before day 15, NPR use was significantly associated with a higher probability of both viral rebound (19.1% vs 7.2%; *P*<.001) and symptom rebound (47.7% vs 16.9%; *P*<.001) at study completion.

The estimates in our study are comparable to other studies, which have reported cumulative incidence of viral rebound in NPR treatment populations from 4% to 27%, and symptom rebound from 0.8% to 32% [21-23]. Studies with lower estimates tended to be secondary analyses of randomized controlled trials or retrospective analyses of electronic medical record data, which may only capture more severe cases [12,22-26]. Studies with higher estimates tended to use more sensitive measures of viral load, and in some cases, they did not require full resolution of symptoms for participants to be eligible for symptom rebound [21,27]. A strength of our study is that it was designed with rebound as the primary outcome and used frequent telehealth, proctor-verified home testing, and symptom surveys. Furthermore, in contrast to most other studies, we did not exclude participants who took partial doses of NPR, which was more common than taking the full 5-day prescription (n=226, 33.7% none, n=246, 36.7% partial, and n=197, 29.4% full), allowing for more accurate real-world estimates of rebound.

We found minimal overall differences in time to initial viral or symptom clearance between the NPR and control groups, suggesting that further investigation was needed into the discordance between how NPR reduces the potency of the virus to ultimately reduce the progression to severe COVID-19, as found in previous studies, and the identified increased rates of rebound or persistent virus. Time to initial symptom clearance was longer than viral clearance, consistent with another prospective study [21]. The tendency for symptoms to remain after testing negative was further seen in examinations of consistency between positive tests and symptoms across the 15-day acute phase follow-up (Table S6 in Multimedia Appendix 1). Of note, we captured self-reported presence or absence of symptoms, whereas symptom severity might yield additional insights.

An additional finding from our study is that at 5 days after enrollment (corresponding to up to 7 days after first turning positive), more than one-third (244/669, 36.5%) of participants remained positive on rapid antigen tests (Figure 3). Although Centers for Disease Control and Prevention guidance has largely recommended ending isolation after 5 days, our findings suggest that individuals may remain infectious for longer and should therefore remain cautious.

Numerous mechanisms have been hypothesized for why rebound occurs. The constant push and pull between virus and immunity drives “predator-prey dynamics,” where the virus’ presence triggers the activation of immune defenses, and virus clearance signals these immune defenses to shift toward a recovery mode [28]. If the shift is too early, the relaxed defenses can allow remaining virus to rapidly re-expand. Regarding NPR and increased rebound risk, it is possible that early treatment initiation pharmaceutically drives down virus growth while abrogating the need to raise a robust immunological defense. Thus, upon removal of NPR, remaining virus could replicate at a rate that outpaces immune defenses, leading to a virus rebound until defenses catch up. Consistent with many COVID-19 symptoms in an era of widespread immunity that represent activating immune defenses (ie, fever and congestion), rebound symptoms generally reflect mounting immune defenses. If immunity must increase to clear residual virus after completion of NPR treatment, many individuals would be expected to experience symptoms; this would lead to a more common symptom rebound phenomenon and a less common full virus rebound, which aligns with our results [29-31].

The COVID-19 Rebound Study was not designed to investigate the efficacy of NPR. Prior studies clearly demonstrate the impact of NPR on reducing COVID-19–related hospitalizations and severe outcomes [3-7,15]. On the basis of our rebound-related findings, our recommendation to clinical health care providers prescribing NPR is to inform patients of the higher likelihood of viral and symptom rebound, that there is insufficient evidence regarding the severity of these symptoms, and that the evidence supporting reduction in hospitalizations and progression to severe COVID-19 is strong.

This was a nonrandomized study that may be limited by selection bias, recall bias, and an unbalanced sample size. While NPR was the primary COVID-19 oral antiviral therapeutic, we did not systematically capture use of emerging alternative antiviral therapies in the control group, which may influence rebound estimates. Our definition of symptom rebound (≥1 symptom after resolution) is sensitive but may have lacked specificity, potentially overestimating clinically meaningful rebound events. Another limitation is that we required enrollment within 48-hours of a positive rapid antigen test; however, participants may have had symptoms and disease for longer durations prior to self-testing and/or asymptomatic participants may have tested at an unknown time point after infection, thereby leading to underestimates on time to viral or symptom clearance. Timing estimates were also impacted by the length of the study as not all participants experienced clearance by day 15. The relatively large numbers of participants in both groups helps to mitigate, though not necessarily eliminate, the impact of these potential biases as they pertain to relative differences between groups. The social media recruitment arm was opened partway through the study primarily to increase the number of participants who were not intending to take NPR, as those recruited through the eMed platform may have been drawn to the test-to-treat virtual care paradigm. The analysis excluded 27% (248/917) of consented participants due to missing data that precluded capture of potential rebound. Excluded participants tended to be less vaccinated, NPR users, and non-White (Table S7 in Multimedia Appendix 1). There was also nonresponse or dropout during the study, whereby 67.6% (452/669) of the analysis sample completed all acute study phase surveys.

### Conclusions

In summary, this prospective observational study of COVID-19–positive outpatients showed that NPR treatment decision was associated with a substantially increased risk of both viral and symptom COVID-19 rebound. No difference was found in the overall time to initial clearance of viral test positivity or symptoms between the NPR and control groups, although the NPR group was older and had more preexisting conditions at baseline. With this important caveat for interpretation of our findings, there is a clear-cut need for more durable antiviral therapies or regimens and for improved knowledge regarding optimal patient selection.

## Supplementary material

10.2196/80263 Multimedia Appendix 1 The figures show time to initial viral and symptom clearance, time from clearance to rebound, and duration of rebound. The tables show differences between the two outreach cohorts, sensitivity analyses of 15-day COVID-19 cumulative incidence estimates, Cox proportional hazard regressions for symptom rebound, congruence of viral and symptom reports, and characteristics of participants included vs excluded from analyses.

## Abbreviations

CLIA: Clinical Laboratory Improvement Amendments
NPR: nirmatrelvir plus ritonavir
REDCap: Research Electronic Data Capture
SRTI: Scripps Research Translational Institute
STROBE: Strengthening the Reporting of Observational Studies in Epidemiology
