# Supplementary material for: COVID-19 Rebound in Nirmatrelvir Plus Ritonavir Treatment and Control Groups: Prospective Cohort Study
Source: Interact J Med Res. 2026 Jun 2;15:e80263. doi: 10.2196/80263 (PMC13229391; doi:10.2196/80263)
Supplement: Multimedia Appendix 1 [file ijmr-v15-e80263-s001.docx]

**Supplementary Material**

**Supplementary Figures**

**
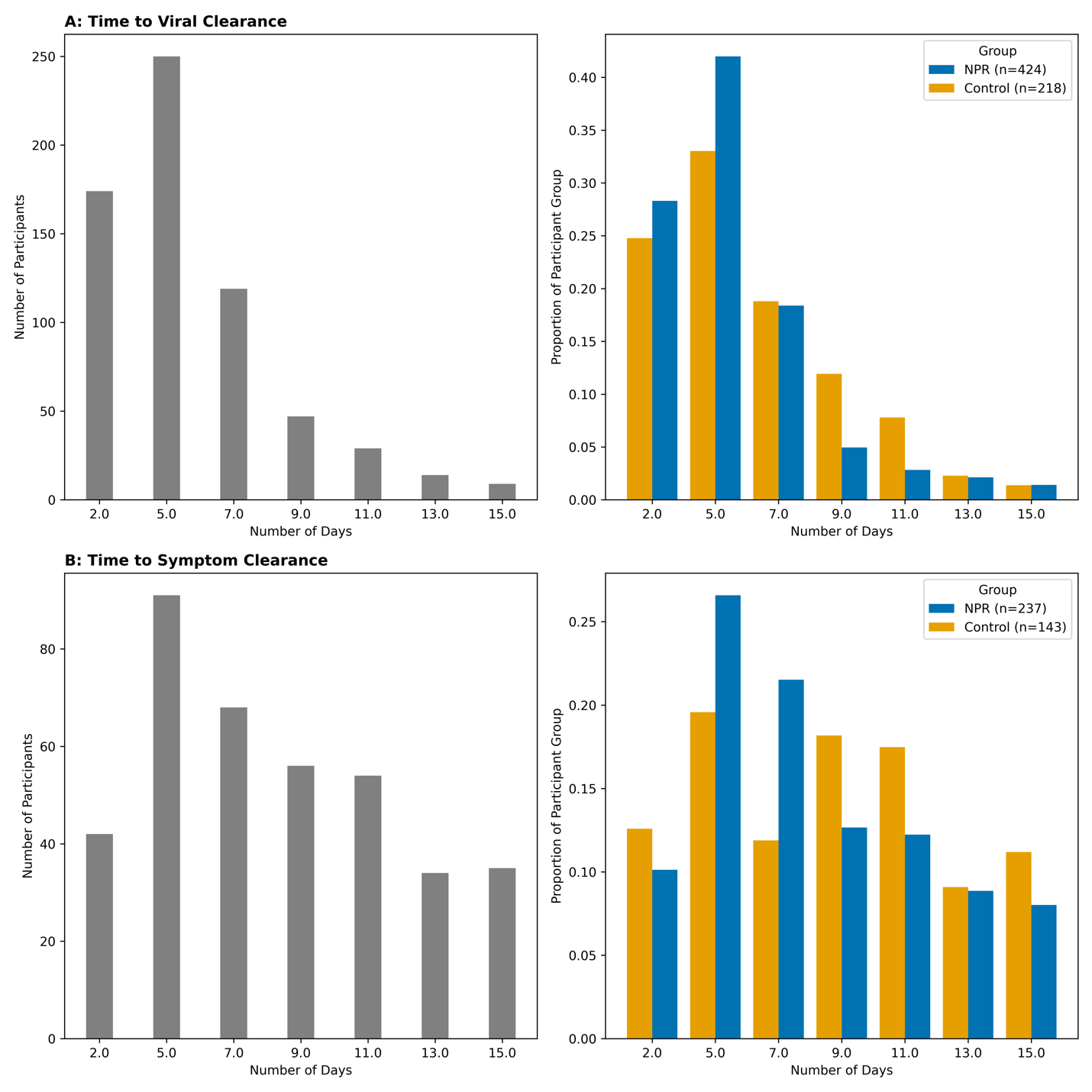
**

**Figure S1 Time from study enrollment to initial clearance.** A: Time to first negative rapid antigen test among the subset of participants who reached viral clearance within the 15-day acute phase, overall (left) and by study group (right). B: Time to initial report of no symptoms among the subset of participants who reported symptom clearance within the 15-day acute phase, overall (left) and by study group (right).

**
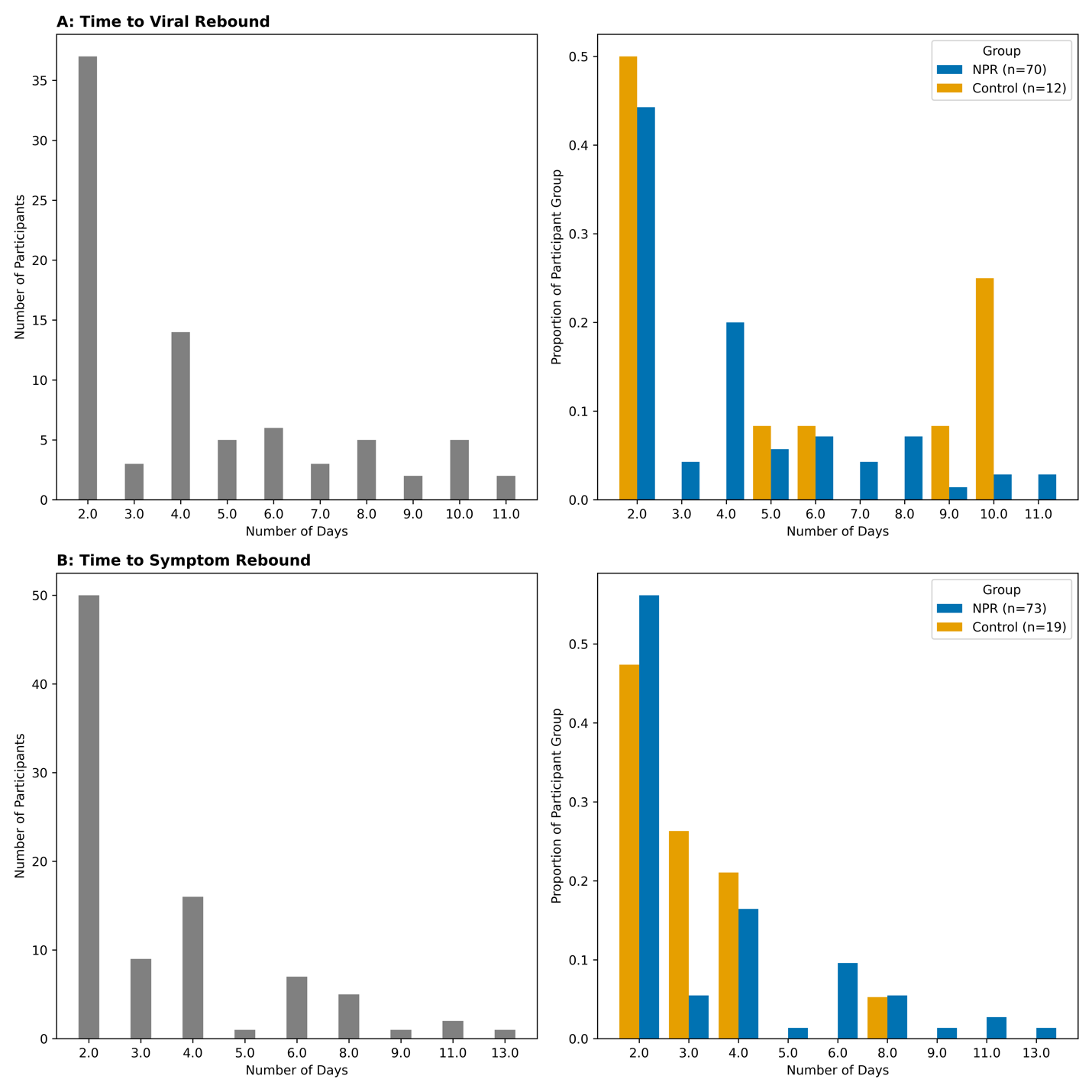
**

**Figure S2 Time from initial clearance to rebound.** A: Time from first negative to rebound positive rapid antigen test among the 82 participants who experienced viral rebound within the 15-day acute phase, overall (left) and by study group (right). B: Time from initial report of no symptoms to report of at least one rebound symptom among the 92 participants who experienced symptom rebound within the 15-day acute phase, overall (left) and by study group (right).

**
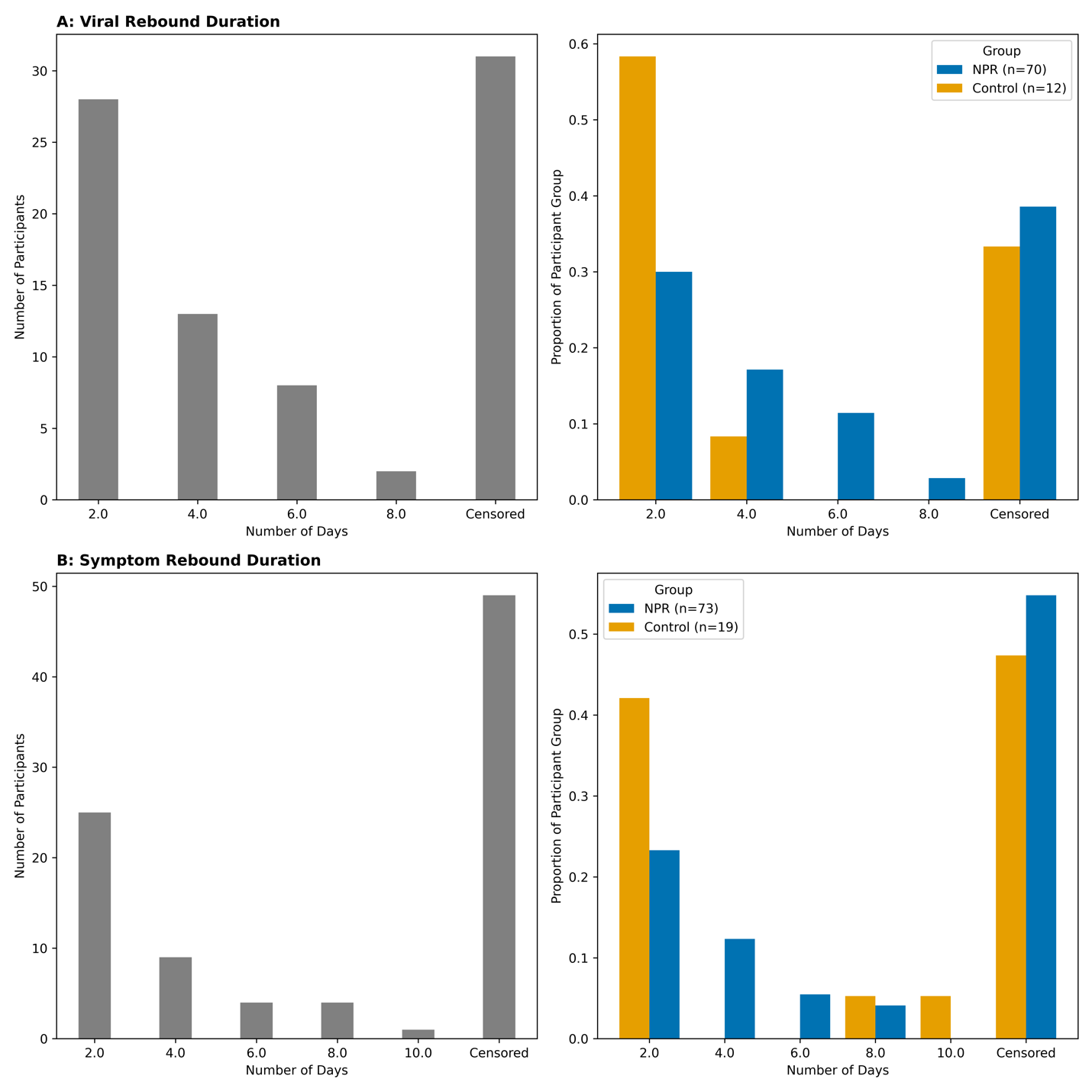
**

**Figure S3 Duration of rebound.** A: Time from rebound positive test to subsequent negative rapid antigen test overall (left) and by study group (right). B: Time from rebound symptoms to subsequent report of zero symptoms overall (left) and by study group (right). The censored bar includes participants that did not clear rebound virus or symptoms by Day 15 or were censored for missingness after rebound.

**Supplementary Tables**

|  |  | **Overall** | **Cohort 1** | **Cohort 2** | **P value** |
| --- | --- | --- | --- | --- | --- |
| **Number of Participants (n)** | | 669 | 251 | 418 |  |
| **Treatment Group, n (%)** | |  |  |  |  |
|  | Control | 226 (33.8) | 70 (27.9) | 156 (37.3) |  |
|  | NPR | 443 (66.2) | 181 (72.1) | 262 (62.7) |  |
| **15-Day Cumulative Rebound, n (%)** | |  |  |  |  |
|  | Viral | 82 (12.3) | 37 (14.7) | 45 (10.8) |  |
|  | Symptom | 92 (13.8) | 40 (15.9) | 52 (12.4) |  |
| **Age, n (%)** | |  |  |  | .26 |
|  | 18-44 | 344 (51.4) | 113 (45.0) | 231 (55.3) |  |
|  | 45-64 | 258 (38.6) | 109 (43.4) | 149 (35.6) |  |
|  | > 65 | 67 (10.0) | 29 (11.6) | 38 (9.1) |  |
| **Sex, n (%)** | |  |  |  | .36 |
|  | Female | 419 (62.6) | 145 (57.8) | 274 (65.6) |  |
|  | Male | 249 (37.2) | 106 (42.2) | 143 (34.2) |  |
|  | Intersex | 1 (0.1) | 0 (0.0) | 1 (0.2) |  |
| **Race, n (%)** | |  |  |  | .26 |
|  | American Indian or Alaska Native | 2 (0.3) | 2 (0.8) |  |  |
|  | Asian | 42 (6.3) | 10 (4.0) | 32 (7.7) |  |
|  | Black, African American, or African | 41 (6.1) | 22 (8.8) | 19 (4.5) |  |
|  | Hispanic, Latino, or Spanish | 47 (7.0) | 21 (8.4) | 26 (6.2) |  |
|  | White | 491 (73.4) | 181 (72.1) | 310 (74.2) |  |
|  | Multiple | 33 (4.9) | 10 (4.0) | 23 (5.5) |  |
|  | Decline to Answer | 10 (1.5) | 5 (2.0) | 5 (1.2) |  |
|  | Other or Unknown | 3 (0.4) | 0 (0.0) | 3 (0.6) |  |
| **COVID-19 vaccine, n (%)** | | 637 (95.2) | 236 (94.0) | 401 (95.9) | .75 |
|  | At least one | 637 (95.2) | 236 (94.0) | 401 (95.9) | .75 |
| **Preexisting Conditions, n (%)** | | | | | |
|  | Asthma | 117 (17.5) | 36 (14.3) | 81 (19.4) | .384 |
|  | Autoimmune Condition | 63 (9.4) | 19 (7.6) | 44 (10.5) | .688 |
|  | Cancer | 35 (5.2) | 14 (5.6) | 21 (5.0) | >.99 |
|  | Chronic Bronchitis | 10 (1.5) | 3 (1.2) | 7 (1.7) | >.99 |
|  | COPD | 3 (0.4) | 2 (0.8) | 1 (0.2) | .896 |
|  | Diabetes | 44 (6.6) | 17 (6.8) | 27 (6.5) | >.99 |
|  | Emphysema | 1 (0.1) | 1 (0.4) | 0 (0.0) | .75 |
|  | Heart Disease | 21 (3.1) | 6 (2.4) | 15 (3.6) | .896 |
|  | Heart Failure | 3 (0.4) | 1 (0.4) | 2 (0.5) | >.99 |
|  | High Blood Pressure | 132 (19.7) | 63 (25.1) | 69 (16.5) | .144 |
|  | Other Lung Condition | 7 (1.0) | 2 (0.8) | 5 (1.2) | >.99 |
|  | None | 340 (50.8) | 127 (50.6) | 213 (51.0) | >.99 |
| **Acute Phase Symptom Surveys, mean (SD)** | |  |  |  |  |
|  | Systemic Symptoms | 3.5 (2.4) | 3.4 (2.3) | 3.6 (2.4) | .25 |
|  | Respiratory Symptoms | 4.7 (2.4) | 4.8 (2.4) | 4.6 (2.4) | .25 |
|  | Gastrointestinal Symptoms | 1.0 (1.4) | 0.9 (1.3) | 1.1 (1.5) | .25 |
|  | Neurological Symptoms | 0.4 (1.0) | 0.4 (0.9) | 0.4 (1.1) | .29 |
|  | Other Symptoms | 0.4 (0.9) | 0.2 (0.7) | 0.4 (1.1) | .02 |

**Table S1 Secondary analysis to compare the two study cohorts**. Cohort 1 was recruited from August 2022 to August 2023; Cohort 2 was recruited from August 2023 to December 2023. Tasks for data used in this study were the same between the two cohorts, but Cohort 2 had additional blood sample and test swab tasks that will be presented in a follow-up report. *Legend:* n = number of participants, NPR = Nirmatrelvir Plus Ritonavir; SD = standard deviation.

|  | **15-Day** | **15-Day Conditional** | **No Missing Responses** | **Censor at First Missing** | **11-Day** | **11-Day Conditional** |
| --- | --- | --- | --- | --- | --- | --- |
| **Viral Test Rebound** | | | | | | |
| **Overall** |  |  |  |  |  |  |
| n Eligible | 669 | 633 | 452 | 611 | 669 | 590 |
| n Rebound | 82 | 82 | 68 | 74 | 66 | 66 |
| Proportion | 0.123 | 0.13 | 0.15 | 0.121 | 0.099 | 0.112 |
| **NPR** |  |  |  |  |  |  |
| n Eligible | 443 | 418 | 307 | 405 | 443 | 397 |
| n Rebound | 70 | 70 | 59 | 64 | 60 | 60 |
| Proportion | 0.158 | 0.167 | 0.192 | 0.158 | 0.135 | 0.151 |
| **Control** |  |  |  |  |  |  |
| n Eligible | 226 | 215 | 145 | 206 | 226 | 193 |
| n Rebound | 12 | 12 | 9 | 10 | 6 | 6 |
| Proportion | 0.053 | 0.056 | 0.062 | 0.049 | 0.027 | 0.031 |
| **Symptom Rebound** | | | | | | |
| **Overall** |  |  |  |  |  |  |
| n Eligible | 664 | 345 | 449 | 607 | 664 | 257 |
| n Rebound | 92 | 92 | 69 | 79 | 69 | 69 |
| Proportion | 0.139 | 0.267 | 0.154 | 0.13 | 0.104 | 0.268 |
| **NPR** |  |  |  |  |  |  |
| n Eligible | 440 | 218 | 306 | 403 | 440 | 168 |
| n Rebound | 73 | 73 | 56 | 64 | 56 | 56 |
| Proportion | 0.166 | 0.335 | 0.183 | 0.159 | 0.127 | 0.333 |
| **Control** |  |  |  |  |  |  |
| n Eligible | 224 | 127 | 143 | 204 | 224 | 89 |
| n Rebound | 19 | 19 | 13 | 15 | 13 | 13 |
| Proportion | 0.085 | 0.15 | 0.091 | 0.074 | 0.058 | 0.146 |

**Table S2 Sensitivity analyses on 15-day COVID-19 cumulative incidence estimates.** *The 15-Day* estimate is the primary outcome in the main text, considering all rebound events across the 15-day acute phase regardless of missing data between surveys/tests. *The 15-Day Conditional* Estimate is calculated for the subsample who reached initial clearance before Day 15. *The No Missing Responses* estimate is calculated for the subsample who completed all 8 acute phase surveys and rapid antigen tests. *The Censor at First Missing* only considers surveys up the first missing response, requiring a minimum of three consecutively completed surveys/tests to be eligible for inclusion in incidence calculation. The *11-Day* and *11-Day Conditional* estimates repeat the primary and secondary outcome for 11 instead of 15 days since enrollment. *Legend:* n=Number of participants, NPR = Nirmatrelvir Plus Ritonavir.

|  |  | **Overall** | **Control** | **Partial NPR** | **Full NPR** | **P value** |
| --- | --- | --- | --- | --- | --- | --- |
| **Number of Participants** | | 669 | 226 | 246 | 197 |  |
| **Primary Analysis Group** | |  |  |  |  |  |
|  | Control | 226 (33.8) | 226 (100.0) | |  |  |
|  | NPR | 443 (66.2) | | 246 (100.0) | 197 (100.0) | |
| **Age, n (%)** | |  |  |  |  | .01 |
|  | 18-44 | 344 (51.4) | 146 (64.6) | 103 (41.9) | 95 (48.2) |  |
|  | 45-64 | 258 (38.6) | 69 (30.5) | 108 (43.9) | 81 (41.1) |  |
|  | ≥65 | 67 (10.0) | 11 (4.9) | 35 (14.2) | 21 (10.7) |  |
| **Sex, n (%)** | |  |  |  |  | .55 |
|  | Female | 419 (62.6) | 143 (63.3) | 161 (65.4) | 115 (58.4) | |
|  | Male | 249 (37.2) | 83 (36.7) | 85 (34.6) | 81 (41.1) |  |
|  | Intersex | 1 (0.1) | 0 (0.0) | 0 (0.0) | 1 (0.5) |  |
| **Race, n (%)** | |  |  |  |  | .04 |
|  | AI/AN | 2 (0.3) | 1 (0.4) | 1 (0.4) | 0 (0.0) |  |
|  | AS | 42 (6.3) | 19 (8.4) | 14 (5.7) | 9 (4.6) |  |
|  | BL | 41 (6.1) | 20 (8.8) | 12 (4.9) | 9 (4.6) |  |
|  | HI/LA | 47 (7.0) | 27 (11.9) | 11 (4.5) | 9 (4.6) |  |
|  | WH | 491 (73.4) | 140 (61.9) | 195 (79.3) | 156 (79.2) | |
|  | Multiple | 33 (4.9) | 15 (6.6) | 10 (4.1) | 8 (4.1) |  |
|  | Decline to Answer | 10 (1.5) | 3 (1.3) | 2 (0.8) | 5 (2.5) |  |
|  | Other | 3 (0.4) | 1 (0.4) | 1 (0.4) | 1 (0.5) |  |
| **COVID-19 vaccine, n (%)** | |  |  |  |  |  |
|  | At least one | 637 (95.2) | 208 (92.0) | 237 (96.3) | 192 (97.5) | .08 |
| **Preexisting Conditions, n (%)** | | | | | | |
|  | Asthma | 117 (17.5) | 34 (15.0) | 43 (17.5) | 40 (20.3) | .55 |
|  | Autoimmune Condition | 63 (9.4) | 17 (7.5) | 22 (8.9) | 24 (12.2) | .50 |
|  | Cancer | 35 (5.2) | 5 (2.2) | 18 (7.3) | 12 (6.1) | .12 |
|  | Chronic Bronchitis | 10 (1.5) | 3 (1.3) | 3 (1.2) | 4 (2.0) | .81 |
|  | COPD | 3 (0.4) | 3 (1.3) | 0 (0.0) | 0 (0.0) | .14 |
|  | Diabetes | 44 (6.6) | 10 (4.4) | 20 (8.1) | 14 (7.1) | .50 |
|  | Emphysema | 1 (0.1) | 1 (0.4) | 0 (0.0) | 0 (0.0) | .55 |
|  | Heart Disease | 21 (3.1) | 5 (2.2) | 9 (3.7) | 7 (3.6) | .70 |
|  | Heart Failure | 3 (0.4) | 1 (0.4) | 1 (0.4) | 1 (0.5) | .99 |
|  | High Blood Pressure | 132 (19.7) | 40 (17.7) | 48 (19.5) | 44 (22.3) | .64 |
|  | Other Lung Condition | 7 (1.0) | 1 (0.4) | 3 (1.2) | 3 (1.5) | .64 |
|  | None | 340 (50.8) | 137 (60.6) | 117 (47.6) | 86 (43.7) | .01 |
| **15-Day COVID-19 Rebound, n (%)** | |  |  |  |  |  |
|  | Test Rebound | 82 (12.3) | 12 (5.3) | 37 (15.0) | 33 (16.8) | .002 |
|  | Symptom Rebound | 92 (13.8) | 19 (8.4) | 43 (17.5) | 30 (15.2) | .013 |
| **Acute Phase Symptom Surveys, mean (SD)** | |  |  |  |  |  |
|  | Systemic Symptoms | 3.5 (2.4) | 3.2 (2.2) | 3.3 (2.3) | 4.1 (2.5) | .005 |
|  | Respiratory Symptoms | 4.7 (2.4) | 4.5 (2.4) | 4.7 (2.4) | 4.8 (2.4) | .27 |
|  | Gastrointestinal Symptoms | 1.0 (1.4) | 0.9 (1.3) | 1.0 (1.4) | 1.3 (1.7) | .08 |
|  | Neurological Symptoms | 0.4 (1.0) | 0.3 (0.7) | 0.4 (1.1) | 0.5 (1.3) | .08 |
|  | Other Symptoms | 0.4 (0.9) | 0.3 (0.8) | 0.4 (1.1) | 0.4 (0.9) | .19 |

**Table S3 Secondary analysis to compare baseline characteristics and COVID-19 recovery characteristics by amount of NPR taken**. Participants were grouped by the number of NPR doses they reported taking during the acute study phase into control (0 days), partial NPR (1-4 days), and full NPR (5 days) groups. *Legend:* AI/AN = American Indian or Alaska Native, AS = Asian, BL = African American, HI/LA = Latino, WH = White, n = number of participants, NPR = Nirmatrelvir Plus Ritonavir; SD = standard deviation.

|  | **Hazard Ratio** | **Lower 95%** | **Upper 95%** | **P-Value** |
| --- | --- | --- | --- | --- |
| **NPR** | 2.48 | 1.31 | 4.69 | 0.01 |
| **Age (years)** | 1.01 | 0.99 | 1.03 | 0.34 |
| **Number of Vaccines (count)** | 1.36 | 1.13 | 1.64 | <0.005 |
| **Preexisting Condition: Cardiovascular** | 0.86 | 0.50 | 1.48 | 0.58 |
| **Preexisting Condition: Lung** | 0.84 | 0.46 | 1.52 | 0.56 |
| **Preexisting Condition: Autoimmune** | 0.87 | 0.41 | 1.84 | 0.71 |
| **Preexisting Condition: Diabetes** | 1.53 | 0.72 | 3.26 | 0.27 |
| **Preexisting Condition: Cancer** | 1.69 | 0.81 | 3.52 | 0.16 |
| **Sex: Male** | 0.91 | 0.56 | 1.47 | 0.70 |
| **Race: Hispanic/Latino** | 1.26 | 0.49 | 3.23 | 0.63 |
| **Race: Asian** | 0.84 | 0.30 | 2.34 | 0.74 |
| **Race: Black** | 1.15 | 0.41 | 3.24 | 0.79 |
| **Race: Multiple** | 0.59 | 0.14 | 2.45 | 0.46 |
| **Race: Other** | 0.58 | 0.08 | 4.20 | 0.59 |

**Table S4 Cox’s proportional hazard regression for viral test rebound.** Secondary analysis to examine whether the association between NPR and viral rebound remains after adjustment for potential confounders. The unadjusted Hazard Ratio for NPR was 3.21 (95% CI: 1.74, 5.93).

|  | **Hazard Ratio** | **Lower 95%** | **Upper 95%** | **P-Value** |
| --- | --- | --- | --- | --- |
| **NPR** | 1.87 | 1.1 | 3.20 | 0.02 |
| **Age (years)** | 1.00 | 0.98 | 1.01 | 0.59 |
| **Number of Vaccines (count)** | 1.30 | 1.08 | 1.55 | <0.005 |
| **Preexisting Condition: Cardiovascular** | 0.94 | 0.56 | 1.57 | 0.81 |
| **Preexisting Condition: Lung** | 0.80 | 0.45 | 1.42 | 0.45 |
| **Preexisting Condition: Autoimmune** | 0.63 | 0.27 | 1.48 | 0.29 |
| **Preexisting Condition: Diabetes** | 1.71 | 0.83 | 3.52 | 0.14 |
| **Preexisting Condition: Cancer** | 1.22 | 0.52 | 2.89 | 0.65 |
| **Sex: Male** | 1.49 | 0.96 | 2.30 | 0.07 |
| **Race: Hispanic/Latino** | 0.52 | 0.16 | 1.71 | 0.28 |
| **Race: Asian** | 0.49 | 0.15 | 1.56 | 0.23 |
| **Race: Black** | 3.56 | 1.85 | 6.87 | <0.005 |
| **Race: Multiple** | 0.41 | 0.10 | 1.69 | 0.22 |
| **Race: Other** | 0.59 | 0.08 | 4.30 | 0.61 |

**Table S5 Cox’s proportional hazard regression for symptom rebound.** Secondary analysis to examine whether the association between NPR and symptom rebound remains after adjustment for potential confounders. Participants reporting no symptoms at baseline were excluded from analysis (n=5). The unadjusted Hazard Ratio for NPR was 2.03 (95% CI: 1.23, 3.37).

|  | **Both Positive** | **Both Negative** | **Test Positive & Symptom Negative** | **Symptom Positive & Test Negative** |
| --- | --- | --- | --- | --- |
| **15-Day Acute Phase Period** | |  |  |  |
| Overall | 1681 (34.75%) | 1172 (24.23%) | 110 (2.27%) | 1874 (38.74%) |
| NPR | 1134 (35.23%) | 725 (22.52%) | 74 (2.3%) | 1286 (39.95%) |
| Control | 547 (33.81%) | 447 (27.63%) | 36 (2.22%) | 588 (36.34%) |
| **Viral Rebound Positive Test Subset** | |  |  |  |
| Overall | 151 (82.51%) | N/A | 32 (17.49%) | N/A |
| NPR | 143 (84.62%) | N/A | 26 (15.38%) | N/A |
| Control | 8 (57.14%) | N/A | 6 (42.86%) | N/A |

**Table S6 Congruence of viral test and symptom reports.** The number of person-days where viral test and symptom reports are positive or negative and whether they agree. *Legend*: NPR = Nirmatrelvir Plus Ritonavir, N/A = Not Applicable.

|  |  | **Overall** | **Excluded** | **Included** | **P-Value** |
| --- | --- | --- | --- | --- | --- |
| **Number of Participants (n)** | | 917 | 248 | 669 |  |
| **Treatment Group, n (%)** | |  |  |  | < 0.01 |
|  | Control | 261 (28.5) | 35 (14.1) | 226 (33.8) | |
|  | NPR | 656 (71.5) | 213 (85.9) | 443 (66.2) | |
| **Cohort Version, n (%)** | |  |  |  | < 0.01 |
|  | 1 | 310 (33.8) | 59 (23.8) | 251 (37.5) | |
|  | 2 | 607 (66.2) | 189 (76.2) | 418 (62.5) | |
| **Age, n (%)** | |  |  |  | 0.02 |
|  | 18-44 | 496 (54.1) | 152 (61.3) | 344 (51.4) | |
|  | 45-64 | 343 (37.4) | 85 (34.3) | 258 (38.6) | |
|  | ≥65 | 78 (8.5) | 11 (4.4) | 67 (10.0) |  |
| **Sex, n (%)** | |  |  |  | 0.86 |
|  | Female | 572 (62.4) | 153 (61.7) | 419 (62.6) | |
|  | Male | 343 (37.4) | 94 (37.9) | 249 (37.2) | |
|  | Intersex | 2 (0.2) | 1 (0.4) | 1 (0.1) |  |
| **Race, n (%)** | |  |  |  | 0.006 |
|  | American Indian or Alaska Native | 5 (0.5) | 3 (1.2) | 2 (0.3) |  |
|  | Asian | 62 (6.8) | 20 (8.1) | 42 (6.3) |  |
|  | Black, African American, or African | 68 (7.4) | 27 (10.9) | 41 (6.1) |  |
|  | Hispanic, Latino, or Spanish | 73 (8.0) | 26 (10.5) | 47 (7.0) |  |
|  | Native Hawaiian or other Pacific | 3 (0.3) | 3 (1.2) | 0 (0.0) | |
|  | White | 633 (69.0) | 142 (57.3) | 491 (73.4) | |
|  | Multiple | 51 (5.6) | 18 (7.3) | 33 (4.9) |  |
|  | Other | 7 (0.8) | 5 (2.0) | 2 (0.3) |  |
|  | Decline to Answer | 15 (1.6) | 4 (1.6) | 11 (1.6) |  |
| **COVID-19 vaccine, n (%)** | |  |  |  |  |
|  | At least one | 852 (92.9) | 215 (86.7) | 637 (95.2) | 0.006 |
| **Preexisting Conditions, n (%)** | |  |  |  |  |
|  | Asthma | 155 (16.9) | 38 (15.3) | 117 (17.5) | 0.86 |
|  | Autoimmune Condition | 85 (9.3) | 22 (8.9) | 63 (9.4) | 0.96 |
|  | Cancer | 40 (4.4) | 5 (2.0) | 35 (5.2) | 0.15 |
|  | Chronic Bronchitis | 12 (1.3) | 2 (0.8) | 10 (1.5) | 0.86 |
|  | COPD | 3 (0.3) | 0 (0.0) | 3 (0.4) | 0.86 |
|  | Diabetes | 75 (8.2) | 31 (12.5) | 44 (6.6) | 0.02 |
|  | Emphysema | 1 (0.1) | 0 (0.0) | 1 (0.1) | >0.99 |
|  | Heart Disease | 26 (2.8) | 5 (2.0) | 21 (3.1) | 0.86 |
|  | Heart Failure | 5 (0.5) | 2 (0.8) | 3 (0.4) | 0.86 |
|  | High Blood Pressure | 178 (19.4) | 46 (18.5) | 132 (19.7) | 0.86 |
|  | Other Lung Condition | 8 (0.9) | 1 (0.4) | 7 (1.0) | 0.86 |
|  | None | 474 (51.7) | 134 (54.0) | 340 (50.8) | 0.86 |

**Table S7 Characteristics of participants included vs excluded from analyses.** *Legend:* n = number of participants, NPR = Nirmatrelvir Plus Ritonavir.
